# Pre-workout Induced Demand Ischemia

**DOI:** 10.7759/cureus.33694

**Published:** 2023-01-12

**Authors:** Miguel A Rodriguez Guerra, Ana P Urena Neme, Mohammed Shaban, Constangela Matos Noboa, Tiffany Trinh

**Affiliations:** 1 Medicine, Montefiore Medical Center, Albert Einstein College of Medicine, New York, USA; 2 Cardiology, Medicina Cardiovascular Asociada, Santo Domingo, DOM; 3 Internal Medicine, BronxCare Hospital Center, Icahn School of Medicine at Mt. Sinai, New York, USA; 4 Emergency Medicine, Montefiore Medical Center, Albert Einstein College of Medicine, New York, USA

**Keywords:** troponinemia, sinus tachycardia, subclinical hypothyroidism, demand ischemia, pre-workout supplement

## Abstract

Pre-workout supplement use has increased in recent years. Multiple side effects and off-labeled substances have been reported. We report a case of a 35-year-old patient who recently started a pre-workout and was found to have sinus tachycardia, elevated troponin, and subclinical hyperthyroidism. The echocardiogram showed normal ejection fraction and no wall motion abnormality. Beta-blockade therapy with propranolol was offered, but she refused, and her symptoms and troponin levels improved after proper hydration within 36 hours. A cautious and accurate assessment of young, fitness-enthusiastic patients experiencing unusual chest pain is essential to identify a reversible cardiac injury and possible off-label substances in over-the-counter supplements.

## Introduction

Multi-ingredient pre-workout supplements (MIPS) are a mix of ingredients thought to elicit a synergistic effect on acute exercise performance and subsequent training adaptations [1]. The safety of MIPS is not necessarily known; however, the popularity of their use has increased in recent years. A recent German study reported the use of pre-workout supplements by 25.8% of young athletes [2]. These supplements generally contain ingredients such as caffeine, creatine, beta-alanine, amino acids, nitric oxide agents, herbs, botanicals, or formulas non-well described on their labels [1]. Unlike medications, which are frequently marketed with testing to demonstrate either efficacy or safety, supplements are only loosely regulated by the Food and Drug Administration (FDA). Serious adverse events have been reported in the medical literature, which raises the question of whether supplement use is a contributing cause [3-5]. We present a young female patient who presented to our emergency department with atypical chest pain and palpitations associated with vomiting and elevated troponin levels after pre-workout supplement usage.

## Case presentation

Our patient is a 35-year-old female with no past medical history, who presented to the emergency department complaining of midsternal chest pain and palpitations that started 45 minutes after she started exercising. The symptoms were also associated with recurrent non-bilious, non-bloody vomiting on four occasions. She recently started to drink pre-workout powder, however, did not remember the name but had a picture of the nutrition facts showing high content of nitric oxide and caffeine, but no iodine content. She denied fever, stiff neck, cough, joint pain, shortness of breath, dysuria, dysgeusia, anosmia, lower extremity swelling, chest pain, abdominal pain, constipation, nausea, or diarrhea. The patient denies using drugs, smoking, or alcohol intake. She also denied a family history of metabolic, endocrine, or cardiac disease.

**Figure 1:**
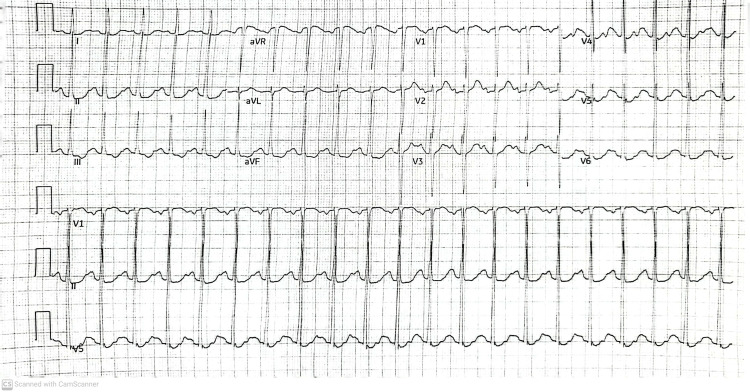
EKG of the patient revealed sinus tachycardia, heart rate 125 bpm, regular rhythm, normal axis, and no pathological ST or T wave changes. EKG: Electrocardiogram

On physical examination, tachycardia was noticed with a heart rate of 118 bpm, a respiratory rate of 16 rpm, a blood pressure of 117/59 mmHg, a temperature of 37C, with an oxygen saturation of 100% on room air. The patient had a body mass index of 25. No pulmonary, cardiovascular, or skeletal abnormality, nor enlarged or swollen lymph nodes were noticed on the physical exam. An electrocardiogram (EKG) did not reveal any acute ischemic changes but showed sinus tachycardia (Figure 1). 

On admission, her laboratory analysis revealed a COVID polymerase chain reaction (PCR) negative result but showed elevated troponin, elevated thyroglobulin antibody (TgAb) 2.0 (<116 IU/mL), and thyroid peroxidase antibody (TPOAb) of 485 (<9 IU/mLco) antibodies, electrolyte imbalance (hypokalemia and hyperglycemia), mild leukocytosis, and anion gap (Table 1). Hypokalemia was addressed in the emergency department. Subclinical hyperthyroidism secondary to possible autoimmune thyroiditis was diagnosed. The patient refused to be medicated with propranolol to reduce her heart rate. After symptomatic therapy and hydration, troponin was downtrending (0.05 ng/mL). Echocardiogram reported an ejection fraction of 60% without wall motion anomaly. Thyroid ultrasonography revealed thyromegaly with diffuse hypervascular changes and heterogeneous echotexture along with no nodules.

**Table 1:** Laboratory results MCV: Mean corpuscular volume, MCH: Mean Corpuscular Hemoglobin, MCHC: Mean corpuscular hemoglobin concentration, RDW-CV: Red cell distribution width-coefficient of variation, CO2: Carbon dioxide, BUN: Blood urea nitrogen, AST: Aspartate aminotransferase, ALT: Alanine transaminase, A/G: Albumin/globulin, GFR: Glomerular filtration rate, Flu: Influenza, RSV: Respiratory syncytial virus, Ig: Immunoglobulin

Hematology		
WBC	12.0	4,500 - 11,000/mm3
RBC	5.05	4.35-5.65 mcL
Hemoglobin	13.7	13.5-17.5 g/dl
Hematocrit	41.1	41-50%
MCV	81.4	80-100μm3
MCH	27.1	25-35 pg/cell
MCHC	33.3	31%-36% Hb/cell
RDW-CV	12.6	11-15%
Platelet count	206	150,000-400,000/mm3
General Chemistry		
Troponin	0.06	<0.04 ng/dL
Sodium, serum	142	136-146 mEq/L
Potassium, serum	2.8	3.5-5.5 mEq/L
Chloride, serum	106	95-105 mEq/L
CO2, serum	19.0	33-45 mm Hg
Total protein	7.0	6.0-7.8 g/dL
Glucose, serum	121	70–100 mg/dL
BUN, serum	17	6 - 24 mg/dl
Creatinine, serum	0.80	0.6–1.2 mg/dL
Alkaline phosphatase, serum	58	25–100 U/L
Bilirubin	0.2	0.1–1.0 mg/dL
Direct bilirubin	0.1	0.0–0.3 mg/dL
AST, serum	22	12–38 U/L
Albumin, SERUM	4.3	3.5-5.5 g/dL
Phosphorous	4.5	3.0-4.5 mg/dL
ALT, serum	30	10–40 U/L
A/G ratio	1.59	1.1-2.5
Uric acid	5.6	3.0-8.2 mg/dL
GFR	81.20	> 60
Magnesium, serum	2	1.5-2.0 mEq/L
Toxicology	negative	
Virology		
SARS COV-2	Positive	
Flu A	Negative	
Flu B	Negative	
RSV	Negative	
Thyroid Function		
Thyroid-stimulating hormone	0001	0.450-5.330 mIU/mL
Free T4	1.26	0.58-1.64 ng/dL
Total T3	140	76-181 ng/dL
Thyroglobulin antibody	2	<1 IU/mL
Thyroid peroxidase antibody	485	<9 IU/mLco
Thyroid stimulating Ig	392	< 140%

**Figure 2:**
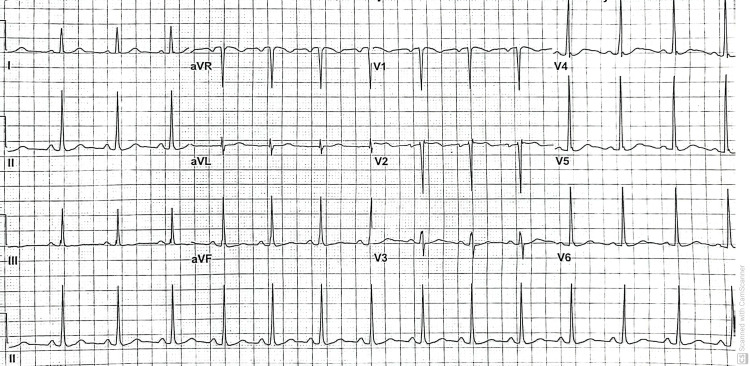
EKG showing normal sinus rhythm, heart rate 86 bpm, normal axis, and no pathological ST or T wave changes. EKG: Electrocardiogram

A second EKG was within normal sinus rhythm (Figure 2). The patient was discharged after three days of hospitalization. During her post-discharge clinic, the patient was asymptomatic and did not have sinus tachycardia.

## Discussion

Multi-ingredient pre-workout supplements are a commonly used mix of caffeine, creatine, beta-alanine, amino acids, and nitric oxide agents. They are not labeled or specified formulas. These combinations are thought to elicit a synergistic effect on acute exercise performance and subsequent training adaptations compared to single ingredients alone [1]. Multi-ingredient pre-workout supplements are believed to impact muscular endurance and subjective mood positively. However, long-term MIPS effects are less clear; but the literature is mainly based on short periods (less than eight weeks). In addition, many formulations may intentionally contain banned substances as ingredients or unintentionally as contaminants which eventually cause health hazards, including cardiac events [6]. Some MIPS with ingredients such as bitter orange, caffeine, nitric oxide products, or sea-calm were reported to be associated with cardiac events [5]. Sibutramine, approved in the US for weight loss in 1997, was withdrawn from the market in 2010 after severe cardiac and vascular complications. Acute fulminant myocarditis associated with ventricular arrhythmia causing sudden cardiac death was reported previously in a healthy patient using an over-the-counter non-labeled sibutramine-containing supplement reported on the autopsy [5].

Caffeine is a cardiovascular stimulant frequently used in weight loss and bodybuilding preparation. However, the caffeine-related sympathetic excitation would cause serious cardiac sequelae up to sudden cardiac death, even in youth [4]. A combination of excessive ingestion of caffeine- and taurine-containing energy drinks and strenuous physical activity can produce myocardial ischemia (MI) by inducing coronary vasospasm; this is why people with preexisting heart conditions are strongly advised to avoid caffeinated MIPS or energy drinks [3,5]. The undisclosed ingredient in pre-workout inducing demand ischemia has been exposed before [7]. Furthermore, severe cardiac health issues were linked to traditional herbs and dietary supplements such as *Angelica sinensis*, *Lycopodium serratum*, and *Tripterygium wilfordii* (Hook F), or the two North American herbs *Caulophyllum thalictroides* and yohimbe [8].

The increased myocardial demand or coronary vasospasm could result in cardiac ischemic events. The diagnosis of acute myocardial infarction is based on changes in cardiac markers, especially troponin levels with or without EKG changes [8]. Myocardial ischemia results from increased myocardial oxygen demand or reduced supply even without acute atherothrombotic plaque disruption [9]. Type 2 MI refers to supply/demand mismatch without acute atherothrombosis [10]. The most common etiologies of type 2 MI are anemia, followed by sepsis, arrhythmia, and post-surgery.

Nitric oxide (NO) is a free radical and oxidative stress marker synthesized through NO synthase in the endothelial cell. It regulates vital functions such as vascular tone, blood flow, mitochondrial respiration, and platelet function [11]. The thyroid hormones affect vascular resistance and endothelial cell function by interacting with membrane ion channels and endothelial NO synthase. Nitric oxide acts on adjacent vascular smooth muscle cells to induce vascular relaxation with net lower systemic vascular resistance. Therefore, when NO availability is reduced, as in hypothyroidism, vascular relaxation is impaired, with resultant increased systemic vascular resistance and endothelial dysfunction. The reverse is hyperthyroidism with net increased peripheral tissue perfusion [12].

Subclinical hyperthyroidism is a common clinical condition leading to mild thyroid dysfunction. Subclinical hyperthyroidism is characterized by apparently 'normal' thyroid hormone (TH) levels and low thyroid-stimulating hormone (TSH) [13]. However, TH levels are higher than the normal set-point of the hypothalamic-pituitary-thalamic axis, resulting in TSH suppression [14]. Many authors prefer the term 'subclinical hyperthyroidism' only for those with entirely suppressed TSH. The latter has the most significant evidence of complications, such as atrial fibrillation and osteoporosis.

Low triiodothyronine (T3) syndrome or non-thyroidal illness syndrome (NTIS) are medical conditions in acute or severe medical illnesses characterized by peripheral and central alterations in the thyroid axis, resulting in low circulating T3 and increased reverse T3 in acutely ill patients. These acute alterations may be mediated by changes in thyroid hormone binding or thyroid hormone uptake by the cell or impaired thyroid hormone release [15]. In many ICU patients, NTIS correlates with a poor prognosis if total thyroxine (TT4) is <4 ug/dL [16]. In both NTI and low T3 syndrome, it is advisable to wait for a period of surveillance and not to act on a single TSH result if the TSH is not entirely suppressed, as a significant proportion of those patients return to normal during follow-up without intervention. On the other hand, prolonged TSH suppression, even if not complete, showed a higher incidence of morbidity in the longer term [17].

Graves' disease is caused by thyroid-stimulating autoantibodies (TSI) to the TSH receptor (TSHR). Hashimoto's thyroiditis is another autoimmune condition caused by thyroid peroxidase and thyroglobulin autoantibodies. Both conditions initially cause hyperthyroidism; however, in some cases, the evolving thyroiditis with tissue damage progress to hypothyroidism status [18]. Thyroid hormones maintain the mitochondrial function to provide the energy needed for diastolic relaxation and systolic myocardial contractility. Hypothyroidism, even subclinical, is proven to worsen ischemic heart disease sequels. This could be explained by endothelial dysfunction resulting from downtrend NO-mediated vascular relaxation. Untreated hyperthyroidism can cause various arrhythmia syndromes and reversible cardiomyopathy through vascular changes, myocyte remodeling, and fibrosis [19,20].

In our case, low TSH could be reflective of an exogenous source of levothyroxine or iodine (possible contamination or ingredient in the pre-workout). Contamination or off-label products in dietary supplements have been reported with major complications, including sudden cardiac death, NTIS, or an evolving subclinical hyperthyroidism pathology. On the other side, TSI (characteristic of Graves' disease) stimulates the thyroid follicles independently, causing more release of thyroid hormone. However, in our case, both total and free T4 were within the normal range; we are not sure if the normal levels of thyroid hormone reflect a direct association between the workout preparation and the positive TSI and thyroid peroxidase and thyroglobulin autoantibodies. If present, this association may directly affect the myocytes causing type 2 MI in our patient. Finally, our patient may have a subclinical thyroid illness manifested by positive autoimmune thyroid markers. Workout preparation that included a possible off-label substance, created a condition that induced circumstances that facilitate demand ischemia in a healthy young person. 

## Conclusions

Multiple products included in pre-workout supplements could predispose to cardiac ischemia. Most of these supplements have a high content of caffeine, nitric oxide, natural stimulants, or off-label products, which have represented numerous complications, including death. Our interesting case presents the importance of being cautious when assessing young fitness-enthusiastic patients experiencing unusual chest pain and exposing the possible presence of off-label products in these supplements. 
